# Dibenzothiophenium Salts: Practical Alternatives to Hypervalent I(III)-Based Reagents

**DOI:** 10.1021/acs.accounts.4c00804

**Published:** 2025-02-03

**Authors:** Manuel Alcarazo

**Affiliations:** Institut für Organische und Biomolekulare Chemie, Georg-August-Universität Göttingen, Tammannstr 2, 37077 Göttingen, Germany

## Abstract

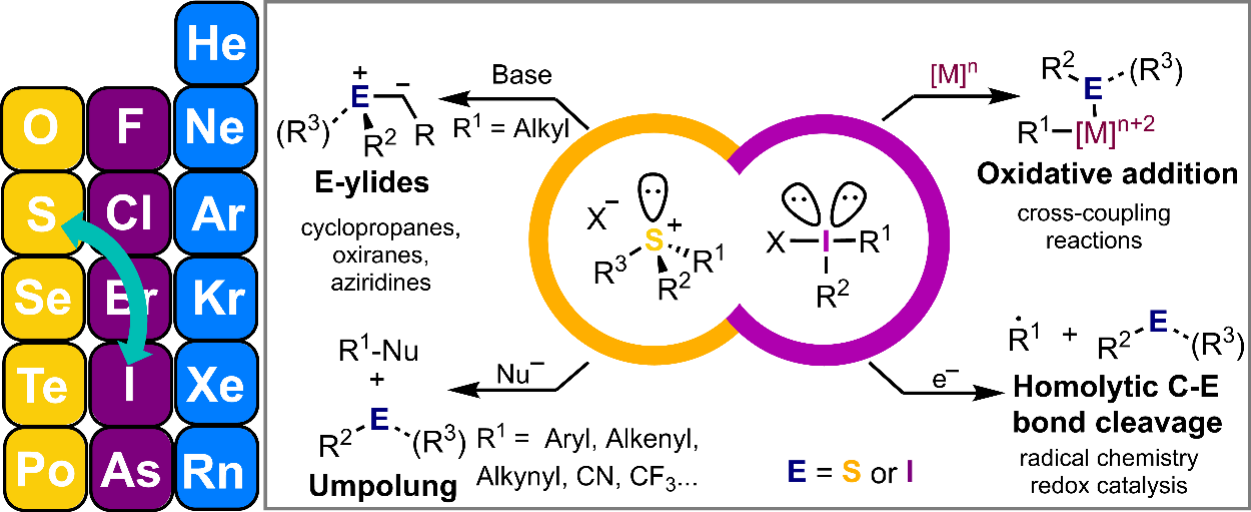

During the past few years, the interest among organic synthesis practitioners in the use of sulfonium salts has exponentially growth. This can arguably be attributed to a series of specific factors: (a) The recent development of more direct and efficient protocols for the synthesis of these species, which make sulfonium reagents of a wide structural variety easily available in multigram scale. (b) The recognition that the reactivity of these salts resembles that of hypervalent iodine compounds, and therefore, they can be used as effective replacement of such species in most of their applications. (c) Their intrinsic thermal stability and tolerance to air and moisture, which clearly surpass that of I(III)-reagents of analogue reactivity, and facilitate their purification, isolation as well-defined species, storage, and safely handling on larger scale. (d) Finally, the possibility to further functionalize sulfonium salts once the sulfur-containing platform has been incorporated. Specifically, this last synthetic approach is not trivial when working with hypervalent I(III)-species and facilitates the access to sulfonium salts with no counterpart in the I(III) realm.

This renewed interest in sulfonium salts has led to the improvement of already existing transformations as well as to the discovery of unprecedented ones; in particular, by the development of protocols that incorporate sulfonium salts as partners in traditional cross-coupling and C–H activation steps or combine them with more modern technologies such as photocatalysis or electrosynthesis. In this Account, the reactivity of a series of sulfonium salts originally prepared in our laboratory will be outlined and compared to their I(III)-counterparts. Some of these reagents are now commercially available, and their use has started to spread widely across the synthetic chemistry community, helping to speed the process of identification of potentially bioactive products or new functionaliced materials. However, challenges still remain. The development of sulfonium reagents characterized by an optimal balance between reactivity and site-selectivity, or showing broader compatibility toward sensitive functional groups is still a need. In addition, the intrinsic stability of sulfonium salts often makes necessary the use of (sophisticated) catalysts that activate the latent reactivity hidden in their structures. Although *a priori* one can see this fact as a disadvantage, it might actually be decisive to harvest the full synthetic potential of sulfonium salts because their thermal stability will surely facilitate the preparation of operational reagents with no counterpart in the context of I(III)-chemistry. If this becomes true, sulfonium salts may contribute to the expediting of retrosynthetic disconnections that, to date, are impossible.

## Key References

HeilmannT.; Lopez-SoriaJ. M.; UlbrichJ.; KircherJ.; LiZ.; WorbsB.; GolzC.; MataR. A.; AlcarazoM.N-(Sulfonio)Sulfilimine Reagents: Non-Oxidizing Sources of Electrophilic Nitrogen Atom for Skeletal Editing. Angew. Chem., Int. Ed.2024, 63, e20240382610.1002/anie.20240382638623698.^1^*This paper describes the one-pot synthesis of λ*^*4*^*-dibenzothiophen-5-imino-N-dibenzothiophenium triflate. Under Rh-catalysis, this reagent efficiently transfers N-sulfonionitrene moieties to the C=C-bond of olefins, forming the corresponding aziridines. Indenes can be transformed into isoquinolines through this method*.

LiX.; GolzC.; AlcarazoM.α-Diazo Sulfonium Triflates: Synthesis, Structure, and Application to the Synthesis of 1-(Dialkylamino)-1,2,3-triazoles. Angew. Chem. Int. Ed.2021, 60, 6943–694810.1002/anie.202014775PMC804847733351262.^2^*The one-pot synthesis of a series of sulfonium salts containing transferable diazomethyl groups is described. Under photochemical conditions diazomethyl radicals are generated, which react with N,N-dialkyl hydrazones to afford 1-(dialkylamino)-1,2,3-triazoles*.

LiX.; GolzC.; AlcarazoM.5-(Cyano)dibenzothiophenium Triflate: A Sulfur-Based Reagent for Electrophilic Cyanation and Cyanocyclizations. Angew. Chem. Int. Ed.2019, 58, 9496–950010.1002/anie.201904557PMC661830031091342.^3^*This contribution describes the efficient umpolung of the cyanide anion using sulfonium chemistry, and the ability of the new reagent developed to promote biomimetic cyanocyclization cascade reactions*.

WaldeckerB.; KraftF.; GolzC.; AlcarazoM.5-(Alkynyl)dibenzothiophenium Triflates: Sulfur-Based Reagents for Electrophilic Alkynylation. Angew. Chem. Int. Ed.2018, 57, 12538–1254210.1002/anie.20180741830063107.^4^*The synthesis and reactivity profile of 5-(alkynyl) dibenzothiophenium triflates are reported in this paper. Many examples are used to illustrate the potential of these salts to become an alternative to the broadly used EBX reagents*.

## 1 Introduction

During the last decades, the increasing demand of structurally complex organic molecules has incentivized the development of mild synthetic protocols that enable the chemoselective manipulation of densely functionalized chemical entities in still useful chemical yields. Such methodologies, which have ended being called “late stage functionalizations” (LSF) approaches, have been intensively implemented in areas such as drug discovery, where the expeditious exploration of the chemical vicinity around an original target compound without the necessity of planning *de novo* syntheses is a must.^5^

It is in that specific context that hypervalent I(III) compounds have aroused as versatile reagents because they make possible the direct C–H functionalization of a broad range of structures, including many (hetero)cycles, through a series of mechanistically differentiated processes. These invariably entail an initial umpolung of the group directly attached to the I(III)-center, and its subsequent release to the desired organic substrate.^6^ The transfer step may occur in many ways; either directly by reaction with an appropriate nucleophile, through the oxidative addition of a low valent metal at the I-X bond (X = C or heteroatom), via one-electron reduction of the I(III)-reagent, which leads to the mesolytic I-X bond fragmentation and the generation of an organic radical, or simply as result of the homolysis of the weak I-X bond.^7^ It is ultimately because of this variety of methods to activate I(III) reagents that the repertoire of carbon–carbon and carbon–heteroatom bond formation processes promoted by such reagents is extremely broad, and includes acylations,^8^ arylations,^9^ alkynylation,^10^ aminations,^11^ fluoroalkylations,^12^ oxidations,^13^ or halogenations^14^ among others. However, and despite this impressive potential, the use of hypervalent I(III)-reagents is often jeopardized by their low thermal stability, which often makes their handling in large scale nontrivial, complicates their purification, and limits their storage possibilities.^15^ It is for these reasons that it urges the identification of alternative families of reagents characterized by an improved balance between reactivity and operational simplicity.

**Figure 1:**
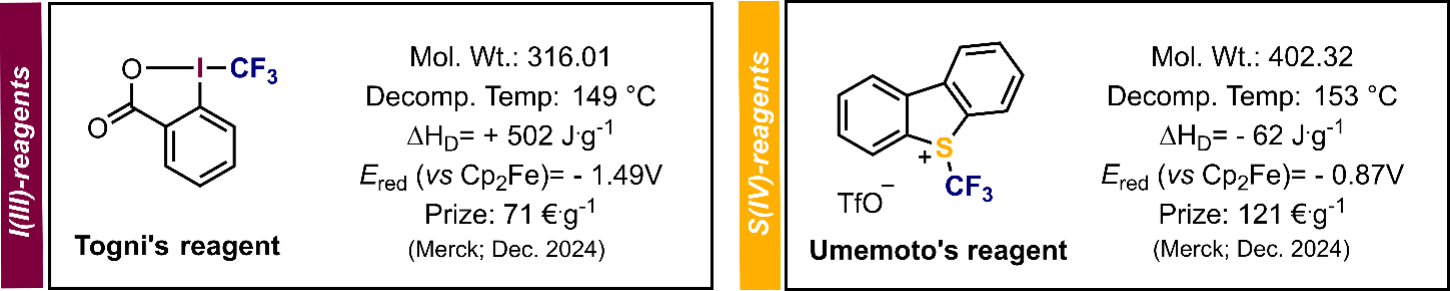
Comparison of the Togni and Umemoto reagents.

Our strategy for designing such reagents was initially inspired by the similar reactivities depicted by the Umemoto and Togni reagents.^16,12c^ Both compounds are widely used for electrophilic and radical trifluoromethylation reactions, but interestingly, they are based on structurally different platforms; a benziodoxol-3-one in the case of the Togni reagent and a dibenzothiophenium unit for the Umemoto one (Figure 1). This formal exchange of the central main group element is not trivial, and has a beneficial impact on the safety profile of the Umemoto reagent.^17^ Differential scanning calorimetry indicates that it decomposes at 153 °C through a slightly endothermic process (Δ*H*_D_ = −67 J·g^–1^), while the decomposition of the Togni reagent at a similar temperature (149 °C) has been determined to be highly exothermic (Δ*H*_D_ = 502 J·g^–1^) and potentially explosive.^17,18^ It is for this reason that already some time ago we decided to evaluate the possibility to further enhance the structural diversity of the transfer reagents based on dibenzothiophenium salts, and to carry out the comparative study of their reactivity with that of I(III) compounds of analogue structure.^19^

This Account outlines our ongoing efforts to design straightforward and high yielding syntheses of dibenzothiophenium salts bearing groups of different nature (-cyano, -alkyne, -arene, -perfluoroalkyl, -diazoalkyl, and -imino) attached to the central S atom, and highlights their reactivity in a series of transformations that range from the umpolung of the hanging moiety to their use for the development of challenging skeletal editing protocols;^20^ thus, exemplifying the advancement of this chemistry over the past decade.^19a,21^ The critical role played by catalysts to achieve the desired reactivities will be emphasized when needed.

## 2 Design and Synthesis of Dibenzotiophenium Salts Containing Novel Functionalities

**Scheme 1:**
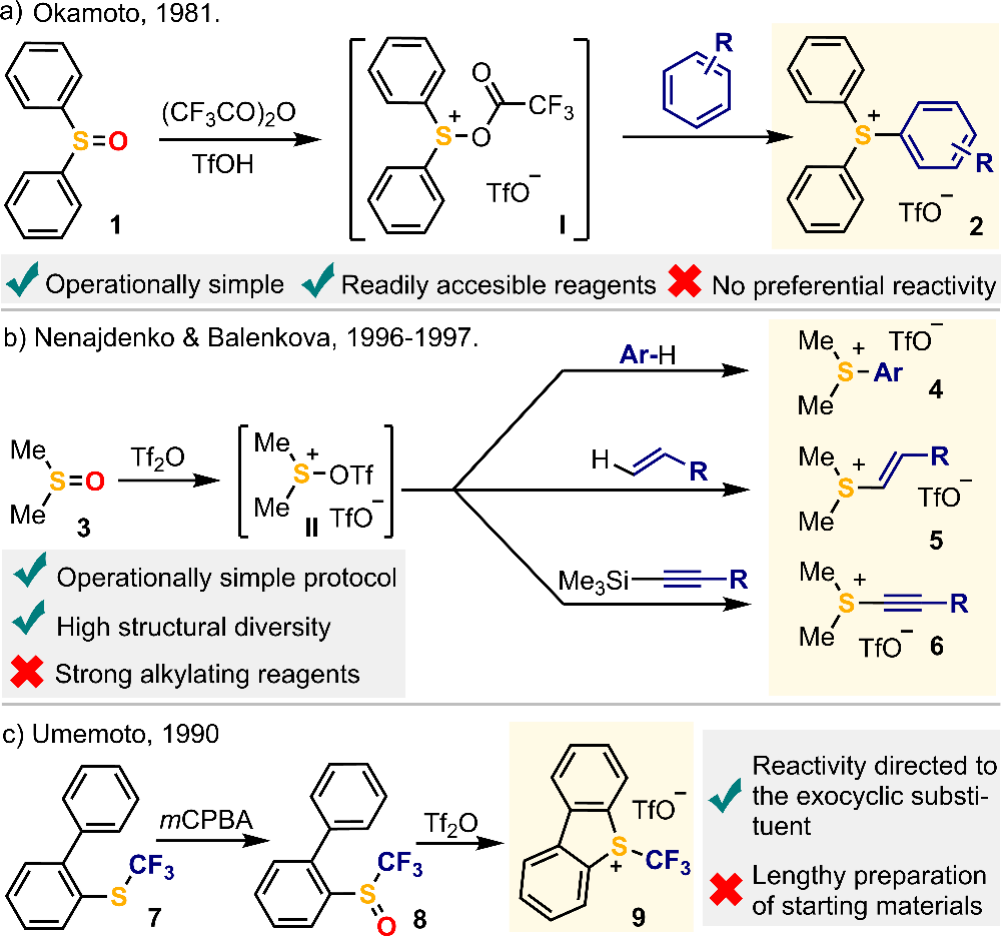
Relevant Advances on the Synthesis of Structurally Diverse Sulfonium Salts

Although pioneering studies on the synthesis and reactivity of sulfonium salts date back more than half a century,^22^ two have been the key improvements regarding their method of synthesis that have made the easy preparation of these reagents in an enormous variety of structures possible. First, the discovery that triarylsulfonium salts could be obtained in excellent yields by in situ activation of diphenyl sulfoxide with anhydrides, and subsequent reaction of the thus prepared acyloxy sulfonium intermediate with arenes (Scheme 1a).^23^ This method is the base for most of the protocols currently used because it offers critical advantages: (a) It does not rely on the alkylation of a sulfide, and (b) the prefunctionalization of the incoming arene is not necessary. Note however that the three aryl substituents of **2** might be transferred in a subsequent reaction *a priori* without selectivity.

Second, the studies of Nenajdenko and Balenkova expanded drastically the scope of the reaction just mentioned. Specifically, these authors prepared dimethylsulfide ditriflate in situ by activation of dimethyl sulfoxide with triflic acid anhydride and showed that this species not only reacts with unfunctionalized arenes but also with alkenes, or TMS-capped alkynes to deliver dimethylaryl-,^24^ dimethylvinyl-,^25^ or dimethylalkynyl-sulfonium salts,^26^ respectively (Scheme 1b). These experiments expanded the diversity of sulfonium salts available; yet, the electrophilic character of the methyl groups in (di)methyl sulfonium salts limits their synthetic utility; nucleophile methylation is often faster than the transfer of the sp^2^- or sp-hybridized substituent.^27^ Other anhydrides such as trifluoroacetic acid anhydride or trifluoromethanesulfonic acid anhydride have been classically used to activate sulfoxides in situ, but for isolation purposes triflic acid anhydride is nowadays the most frequently used reagent by far.^28^

**Scheme 2:**
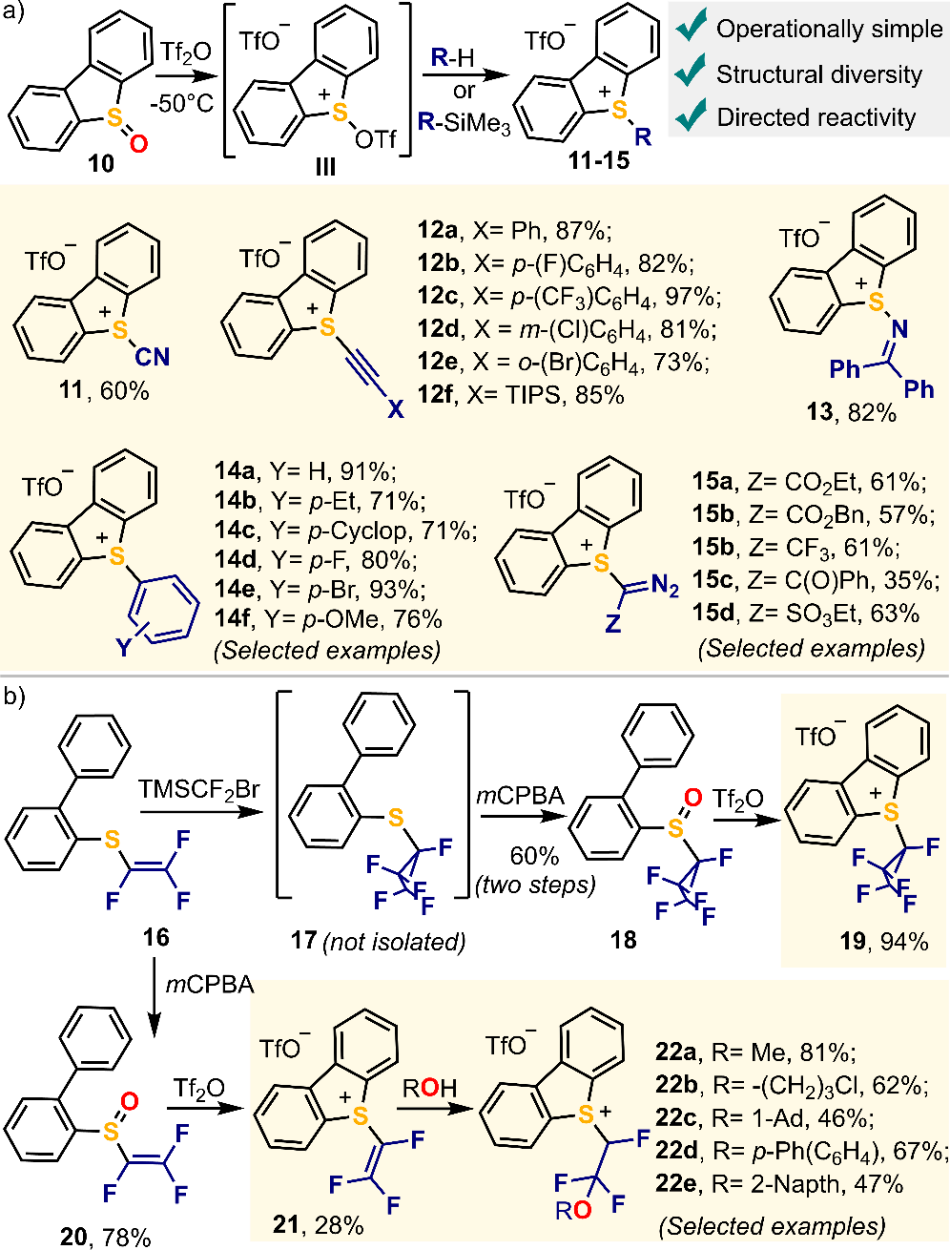
Our Work on the Synthesis of Structurally Diverse Sulfonium Salts

Finally, it is worth mentioning that the intramolecular activation of sulfoxides containing a *o*-biphenyl substituent has also been a widely explored strategy to gain dibenzothiophenium salts.^29^ This is actually the route classically employed for the preparation of the Umemoto reagent,^30^ and although it is long in terms of overall synthetic steps, it is associated with two significant advantages: (a) It allows the attachment of non-nucleophilic groups at the sulfur atom,^31^ and (b) the cyclic architecture of the dibenzothiophenium moiety preferentially directs any further reactivity to the exocyclic S–C bond (Scheme 1c).^32^ It is actually for this fundamental advantage that we decided to privilege the use of the dibenzothiophene platform for the preparation of our reagents; when possible, employing the one-pot strategy based on sulfoxide activation (Scheme 1a-b). Hence, cyano derivative **11**(3) and a series of alkynyl-substituted dibenzothiophenium salts **12a**–**f**^4^ were prepared from TMSCN and the corresponding TMS-capped alkynes, respectively; while imino- **13**,^33^ aryl- **14a**–**f**,^34^ and α-diazo substituted reagents **15a**–**d**^2,35^ have been obtained by C–H sufenylation of the parent nucleophiles. All reactions proceeded in good to excellent yields, and the reagents tolerated purification through classical column chromatography on silica gel. Importantly, **11**–**15** can be stored for moths at room temperature without any special precaution (Scheme 2a).

For the synthesis of fluoroalkylating reagents **19** and **22a**–**e** an Umemoto-type route was followed. Reaction of trifluorovinyl sulfide **16** with TMSCF_2_Br delivers crude pentafluorocyclopropyl-substituted thioether **17**, which was directly submitted to oxidation with *m*-chloroperbenzoic acid without further purification. Treatment of thus obtained sulfoxide **18** with triflic acid anhydride at −45 °C, followed by simple precipitation delivers **19** in 94% yield and analytically pure (Scheme 2b).^36^ Like compounds **11**–**15**, dibenzothiophenium salt **19** is a nonhygroscopic, crystalline, and air-stable solid. Interestingly, compound **16** itself can also undergo treatment with *m*-chloroperbenzoic acid followed by triflic anhydride promoted cyclization to deliver trifluorovinyl reagent **21**; however, this salt slowly decomposes on standing.^37^ This is not the case for its alcohol addition products, **22a**–**e**, which were obtained as air stable compounds after purification through column chromatography in silica gel.

In summary, the selection of the dibenzothiophene platform for our research is due to three fundamental aspects: (a) the aromatic character of this moiety that avoids undesired side reactions, such as alkylations; (b) its cyclic structure that directs the reaction to the exocyclic substituent; and (c) its availability and low price. Other structures also meet these requirements, the most prominent being the thianthrene platform introduced by Ritter.^38^ For the synthesis of sulfonium salts via aromatic C–H sulfenylation, we encourage use of Ritter’s protocol; the *p*-/*m*- and *p*-/*o*- selectivities are superior due to the thermodynamic equilibration to the most stable Wheland intermediate facilitated by the stability of the persistent thianthrene radical cation. On the other hand, our experience indicates that in terms of reactivity the analogy between I(III)-reagents and dibenzothiophenium salts is stronger, probably due to the higher nucleofugality and lower coordination ability of dibenzothiophene.

## 3 Umpolung of Typical Nucleophiles

Nucleophiles often approach a σ-hole at the iodine atom in hypervalent I(III)-reagents; this initial step is usually followed by reductive coupling between the incoming nucleophile and a second substituent already attached to iodine and final release of iodobenzene. Interestingly, in dibenzothiophenium salts the σ*(S-R_exo_) orbital constantly lays at low energy (LUMO or LUMO+1), and it is characterized by a considerable coefficient at the sulfur atom; it is for this reason that very similar mechanistic pathways are facilitated. Note, however, that the nucleofugality of (dialkyl/aryl)sulfides is lower than that or iodoarenes, which often implies a comparatively reduced reactivity in sulfonium-type reagents.^39^

### 3.1 Electrophilic Cyanation

**Figure 2:**
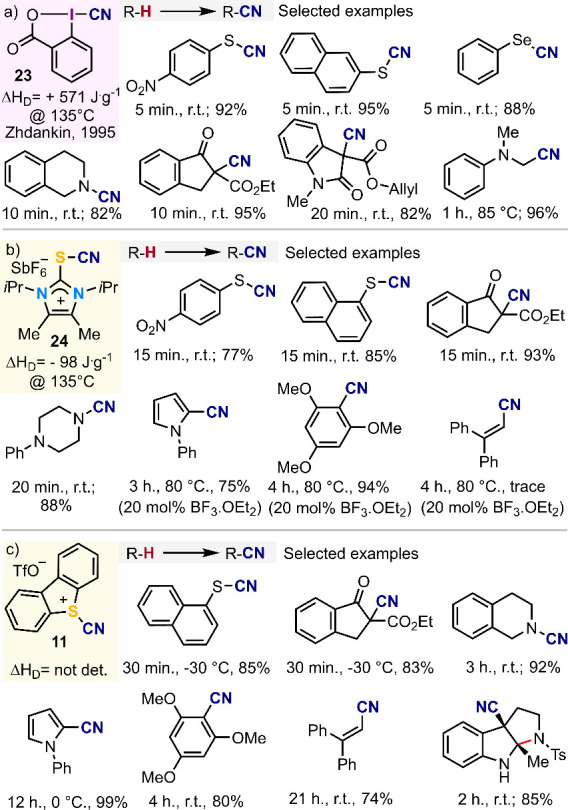
Reactivity comparison between reagents **23**, **24**, and **11**.^48^

The cyano moiety is a privileged precursor of both amines and carbonyl groups,^40^ as well as a functional group commonly present in natural products,^41^ pharmaceuticals,^42^ agrochemicals,^43^ dyes,^44^ and high performance materials.^45^ As it may be expected, most of the protocols reported to incorporate nitrile substituents into organic scaffolds make use of the innate nucleophilicity and easy availability of the cyanide anion,^46^ while the use of strategic disconnections based on formal [CN]^+^ moieties are comparatively scarce.^47^ In this regard a key contribution was done with the introduction of cyanobenziodoxone **23** and derivatives by Zhdankin,^48^ and the subsequent work from Waser^49^ and others,^50^ which demonstrate that the metal-free electrophilic cyanation at nonfunctionalized C-H, N-H, and S-H positions could be efficiently achieved using that reagent or structural derivatives (Figure 2a). This reactivity profile is matched by S-based electrophilic cyanating reagent **24**, a compound that is isolobal to **23** (Figure 2b).^51^ Interestingly, when **24** is activated by BF_3_·OEt_2_, the scope of the electrophilic C–H cyanation could be extended to electron rich aromatic skeletons such as pyrroles, indoles, and anisole derivatives. This reactivity has not been reported using reagent **23.** Probably, the oxidative character of that compound makes it incompatible with electron rich (hetero)aromatic compounds.

In 2019, we also introduced *S*-(cyano) dibenzothiophenium triflate **11** as an efficient [CN]^+^ synthon.^3^ Unfortunately, the direct comparison of its reactivity profile with that of **23** and **24** is not easy because the reactivity reported has not been carried out under identical conditions; yet, some tendencies are clear. We know from our own experience that the electrophilic transfer of the cyano-unit to anilines, thiols, silyl enol ethers, and activated methylene groups are equally successful in terms of isolated yields for the three reagents. It is of note that the C–H cyanation of some electron rich homo-, hetero-, and polycyclic aromatic structures takes place employing **11**, but the substrate scope is broader when **24** is employed in combination with BF_3_·OEt_2_. Unfortunately, our attempts to further activate **11** with the same Lewis acid were unsuccessful and leaded to complex reaction mixtures_._ On the other hand, the intramolecular cyanocyclisation of *N*-protected tryptamine derivatives to deliver a series of cyano-substituted tetrahydropyrroloindoles of potential synthetic and/or pharmacological interest proceeds satisfactorily with **11**; we have not observed this reaction when using **24** as electrophilic cyanating reagent (Figure 2c). Finally, the *N*-alkyl cyanation of *N*,*N*-dialkylanilines, a reaction that efficiently proceeds using cyanobenziodoxone **23**,^48^ has not been reported with **11** probably due to the diminished oxidative character of the sulfonium salt.

### 3.2 Electrophilic Alkynylation

**Scheme 3:**
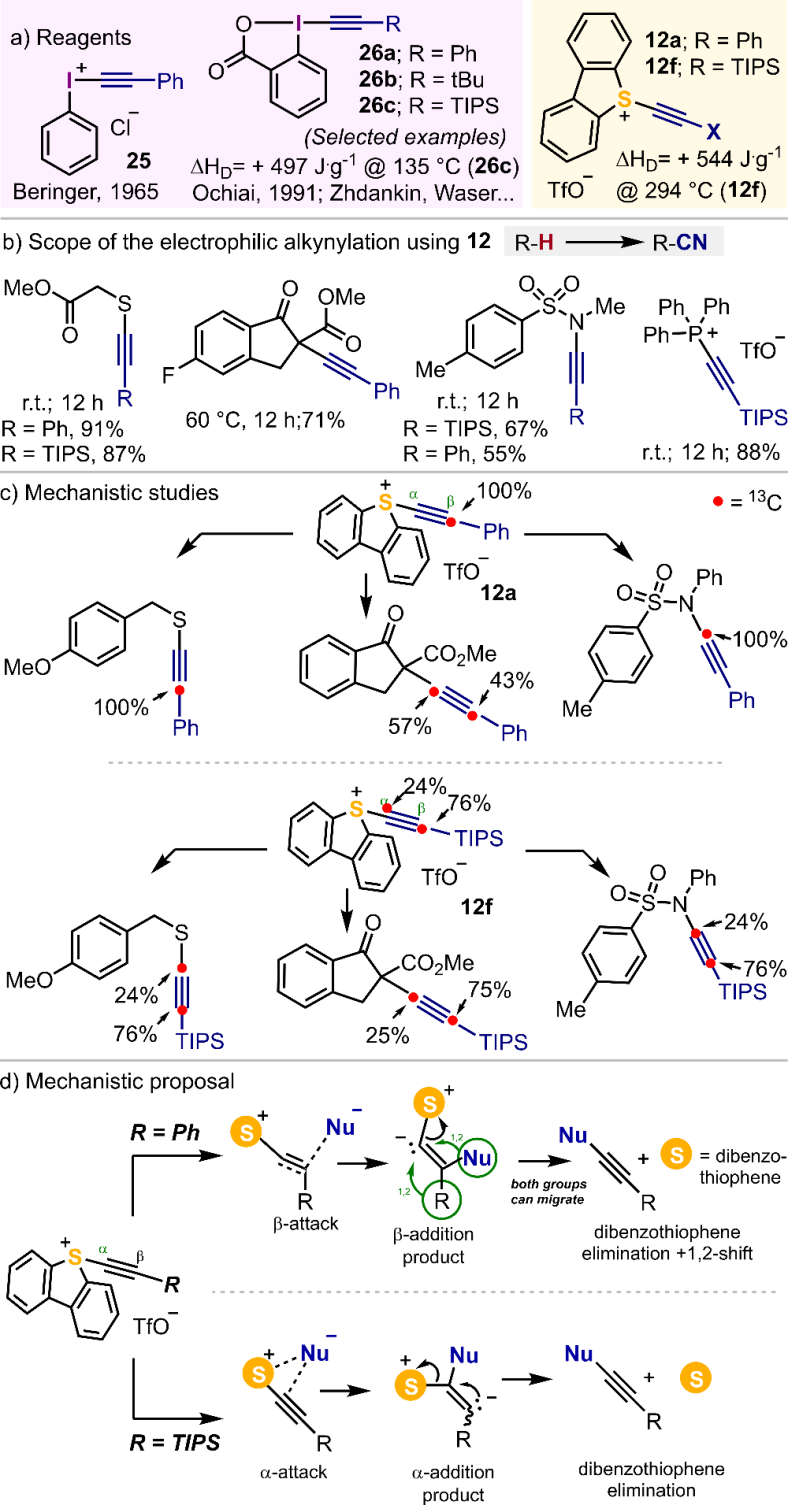
Reactivity of **12** towards Nucleophiles and Mechanistic Studies^52−54^

Alkynyl-substituted hypervalent I(III)-reagents were originally prepared by Beringer as acyclic derivatives **25**(52) and later by Ochiai as benziodoxoles **26**.^53^ This family of reagents has demonstrated huge potential for the electrophilic alkynylation of typical organic nucleophiles; but specifically, widespread applications have been found during the last years on the bioorthogonal functionalization of peptides, and the alkynylation of carbon-centered radicals.^10^ In comparison, *S*-(alkynyl) dibenzothiophenium salts are much less developed. We reported in 2018 that these compounds react with thiols, sulfonamides and activated methylene groups to afford the desired *S*-, *C*-, and *N*-alkynylated products,^4^ and studied the mechanism of these reactions by isotope labeling (Scheme 3a-c). These studies revealed that **12f**, decorated with a terminal TIPS moiety, undergoes an attack by nucleophiles at the α-carbon of the triple bond. Contrarily, for substrate **12a** bearing a terminal Ph-unit we propose that nucleophiles attack at the β-position, followed by elimination of the dibenzothiophene moiety, and subsequent 1,2-migration of one of the groups to the α-carbon (Scheme 3d); however, for the specific case of the thiol nucleophile, we cannot discard its direct attack at the α-carbon of the alkyne moiety. Sulfonium salts **12a**–**f** also react with HX or X_2_ (X = halogen) to deliver β-halovinyl- or α,β-dihalovinyl sulfonium salts, respectively, via regioselective addition to the carbon–carbon triple bond.^54^

**Scheme 4:**
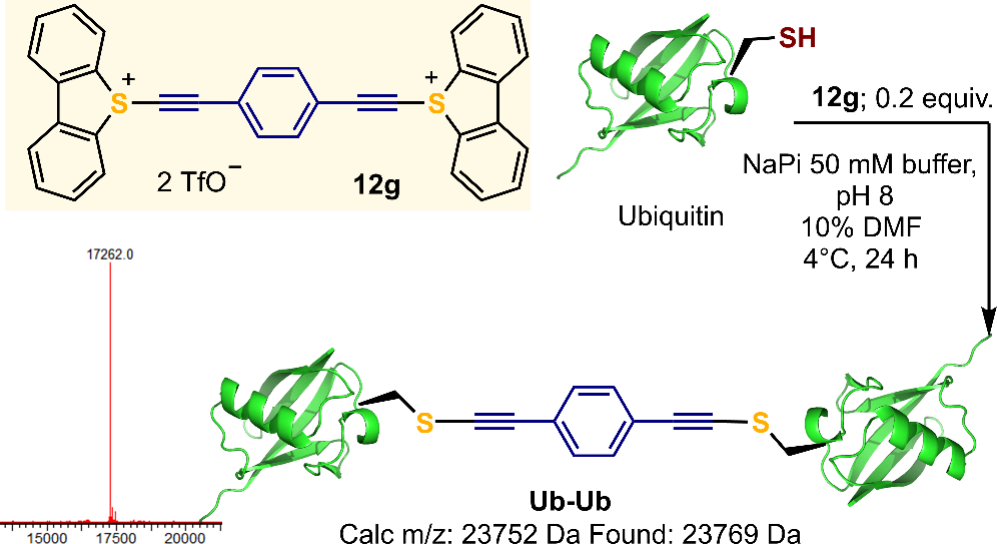
Protein Dimerization by Electrophilic Alkynylation

In cooperation with the Bernardes group, we have also introduced 5-(alkynyl)dibenzothiophenium triflates as reagents to prepare protein conjugates of different structure through site-selective cysteine alkynylation.^55^ The protocol developed allows a highly efficient labeling of free cysteine-containing proteins with relevant biological roles, such as ubiquitin, the C2A domain of Synaptotagmin-I, or HER2 targeting nanobodies. Interestingly, we also designed doubly alkynylating reagent **12g** that allows the access to protein-thiol, protein-peptide and protein–protein conjugates; diubiquitin dimers were also prepared through this approach (Scheme 4).

## 4 Photogeneration of Radicals

The photogeneration of alkyl radicals via single electron transfer to sulfonium salts is a long-time known process. Already in 1979, the desulfuration of phenacyl sulfonium salts was described by Kellogg employing Ru(bpy)_3_Cl_2_ as a photocatalyst, and Hantzch esters as hydrogen atom donors.^56^ That specific model transformation did not have any special synthetic relevance at that time; yet, the methodology employed to generate organic radicals from sulfonium salts places this seminal work at the origin of the recent developments in the area.^21e,56b^ Also for this reactivity mode there is an underlying relation between sulfonium and iodonium salts; in fact, iodonium reagents are equally effective precursors of organic radicals under basically identical reaction conditions.^57^

### 4.1 Aryl Radical Generation: Arylation of (Hetero)arenes

**Scheme 5:**
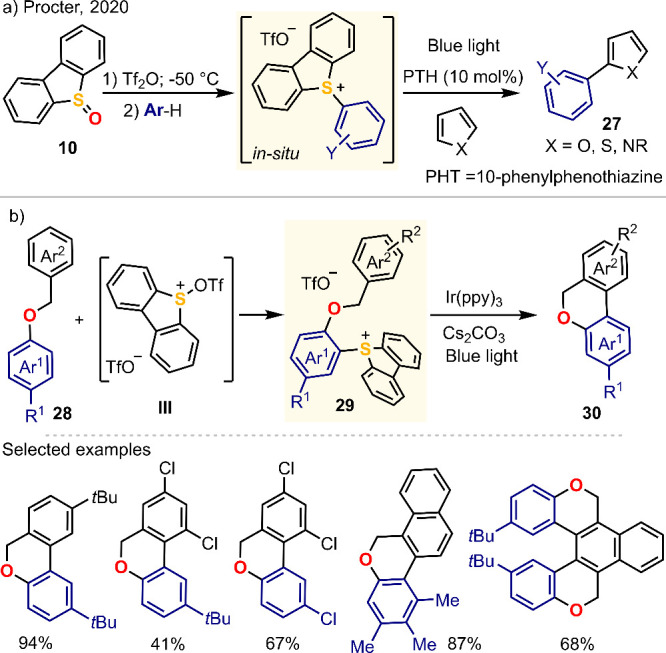
Two-Step 6*H*-Benzo[*C*]chromene Synthesis from Benzyl Ethers^59,60^

In this context, inspired by the previous works of Ollivier on the generation of aryl radicals from triarylsulfonium salts^32b^ and König on the transition metal-free photocatalytic C(sp^2^)-H arylation of (hetero)arenes using aryl diazonium salts,^58^ Procter developed a variant of the arylation reaction that uses aryl dibenzothiophenium salts as electrophilic partners instead.^59^ Far from being trivial, this strategic change significantly impacts the applicability and scope of the transformation because (a) it avoids the handling of explosive diazonium salts and (b) uses dibenzothiophenium salts that are prepared in situ by direct sulfenylation of nonprefunctionalized aromatic feedstocks (Scheme 5a). The proposed photoredox catalytic cycle starts with the formation of the required aryl radical by single-electron reduction from the excited photocatalyst to the sulfonium salt. The aryl radical then undergoes addition to the heteroarene, followed by single-electron oxidation of the resultant heteroaryl radical. This regenerates the photocatalyst, and delivers, after deprotonation, the desired cross-coupled product.

Our contribution to this area has been 2-fold. First, we isolated a complete series of *S*-aryl substituted dibenzothiophenium salts **14a**–**x**,^34^ and determined the scope and site-selectivity of the C–H sulfenylation step. (Hetero)aromatic substrates electron poorer than 1,2-dichlorobenzene are inert to the attack of in situ prepared sulfurane **III**, while too electron rich (hetero)arenes, such as for example *N*-methylindole, promote the reduction of **III** to dibenzothiophene and afford complex reaction mixtures. A series of selected examples that illustrate the scope of the C–H sulfenylation are shown in Scheme 2. We later envisaged the possibility to transform Procter’s coupling approach into an efficient cyclization tool through its application to appropriately designed polyaromatic substrates. As a proof of concept, we developed an efficient protocol for the rapid synthesis of 6*H*-benzo[*c*]chromenes from easily available benzyl ethers (Scheme 5b).^60^ The good to excellent yields often achieved are due to two crucial factors: (a) the high site-selectivity of the sulfenylation step, which is easy to predict using simple concepts such as inductive and resonant effects, and (b) a thorough design of the substrate to enforce the selective cyclization of the transient radical at the desired position.

### 4.2 Generation of Diazomethyl Radicals: Synthesis of 1,2,3-Triazoles

**Scheme 6:**
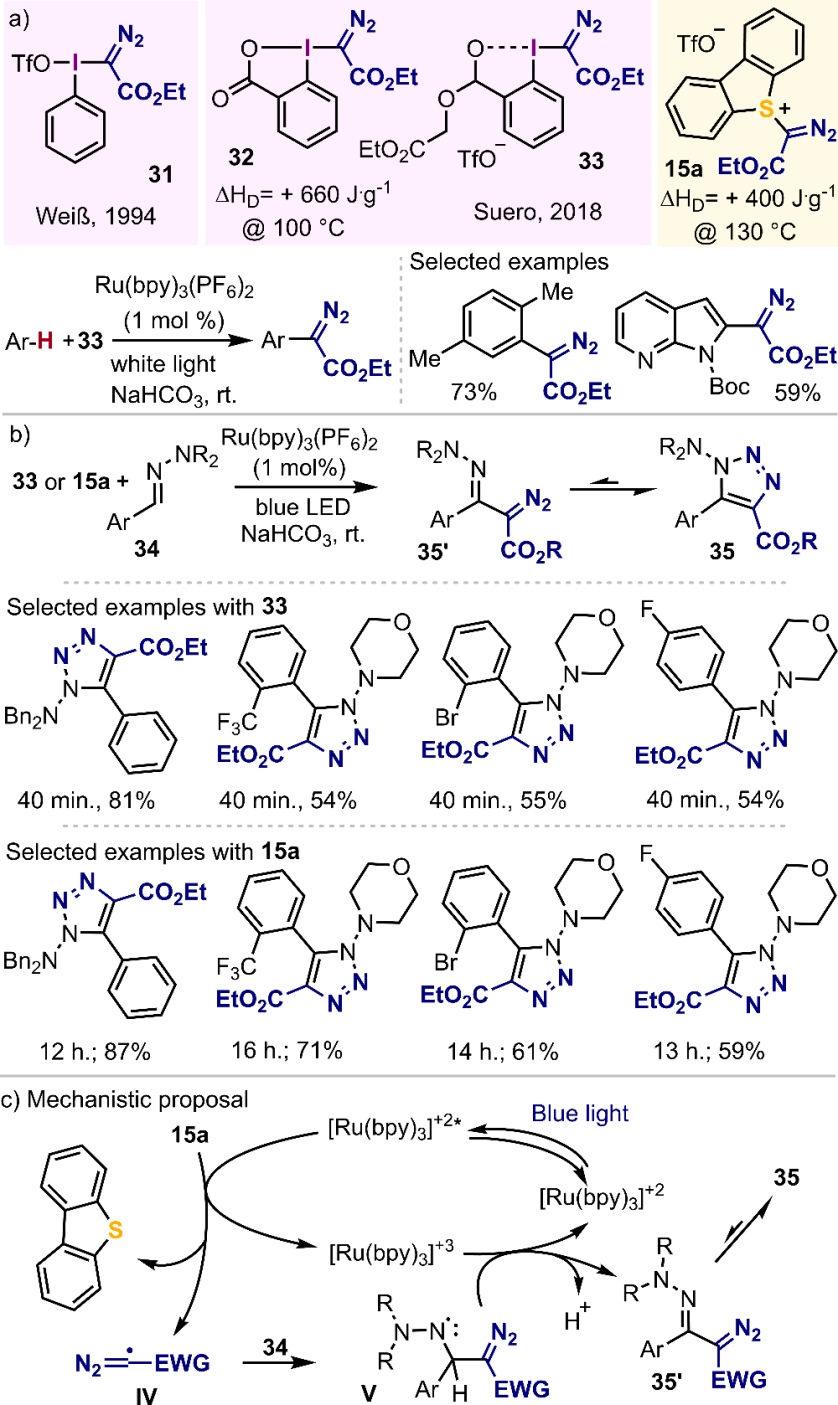
Generation of Diazomethyl Radicals under Photochemical Conditions: Synthetic Applications^61−63^

Although α-diazo-λ^3^-iodanes such as **31** have been known since 1994;^61^ their broad synthetic potential was not fully recognized until Suero in 2018 realized that they could be used as efficient precursors for the generation of diazomethyl radicals **IV** under mild photocatalytic conditions. As result of that discovery, he developed a very original C–H arene diazomethylation reaction by trapping the thus generated diazomethyl radicals with a broad series of structurally diverse (hetero)arenes (Scheme 6a).^62^ Other unsaturated organic nucleophiles have since been used to trap diazomethyl radicals. For example, the reaction with hydrazones delivers 1-amino-1,2,3-triazoles via a formal [3 + 2] cyclization (Scheme 6b).^63^ This transformation is initiated by single-electron transfer to **33** (or **15a**) to generate intermediate **IV**. Subsequent addition to the aldehyde-derived hydrazone **34** forms *N*-centered radical intermediate **V**, which is oxidized by the photocatalyst. Final deprotonation delivers α-diazoalkylhydrazones **35′**. These compounds finally cyclize to 1,2,3-triazole product **35** (Scheme 6b-c).

Interestingly, shortly before the paper of Wang and Li was published,^63^ we reported the same transformation under identical reaction conditions using reagents **15a**–**b**.^2^ Comparison of the available data clearly indicates that the isolated yields of the desired triazoles are, in general, slightly better when sulfonium salt **15a** is utilized as a precursor of the diazomethyl radical; however, reaction times are much longer. This is probably a hint that indicates a more effective radical generation when employing iodine-derived precursor **33**.

### 4.3 Fluoroalkyl Radical Generation: Pentafluorocyclopropanations

Fluorine substituents play a prominent role in the design of drugs and agrochemicals. It is for this reason that these industries continuously demand new protocols that allow the placement of (new) fluorinated moieties on specific positions of the lead structures under optimization.^64^ We recently noticed that despite of the widespread use of the heptafluoroisopropyl (HFIP) group in the context of crop protection,^65^ the pentafluorocyclopropane (PFCP) unit, which formally derives from the cyclization of the two terminal −CF_3_ units in HFIP, had not been used during lead optimization campaigns.^66^ This is probably due to the lack of reagents able to transfer the pentafluorocyclopropane unit. In this context, we recently envisioned that a reagent similar to Umemoto’s but decorated with a C_3_F_5_-moiety instead of the CF_3_-group should be competent to transfer PFCP moieties to arenes via a Minisci-type reaction. Hence, we designed a gram-scale synthesis for **19** and evaluated its reactivity.^36^ Note that there is no analogue of **19** in the I(III)-realm, and the synthesis developed for **19** is not directly transferable to such hypothetical reagents because structural modifications in iodine-based reagents are not straightforward once the I atom is already installed.

**Scheme 7:**
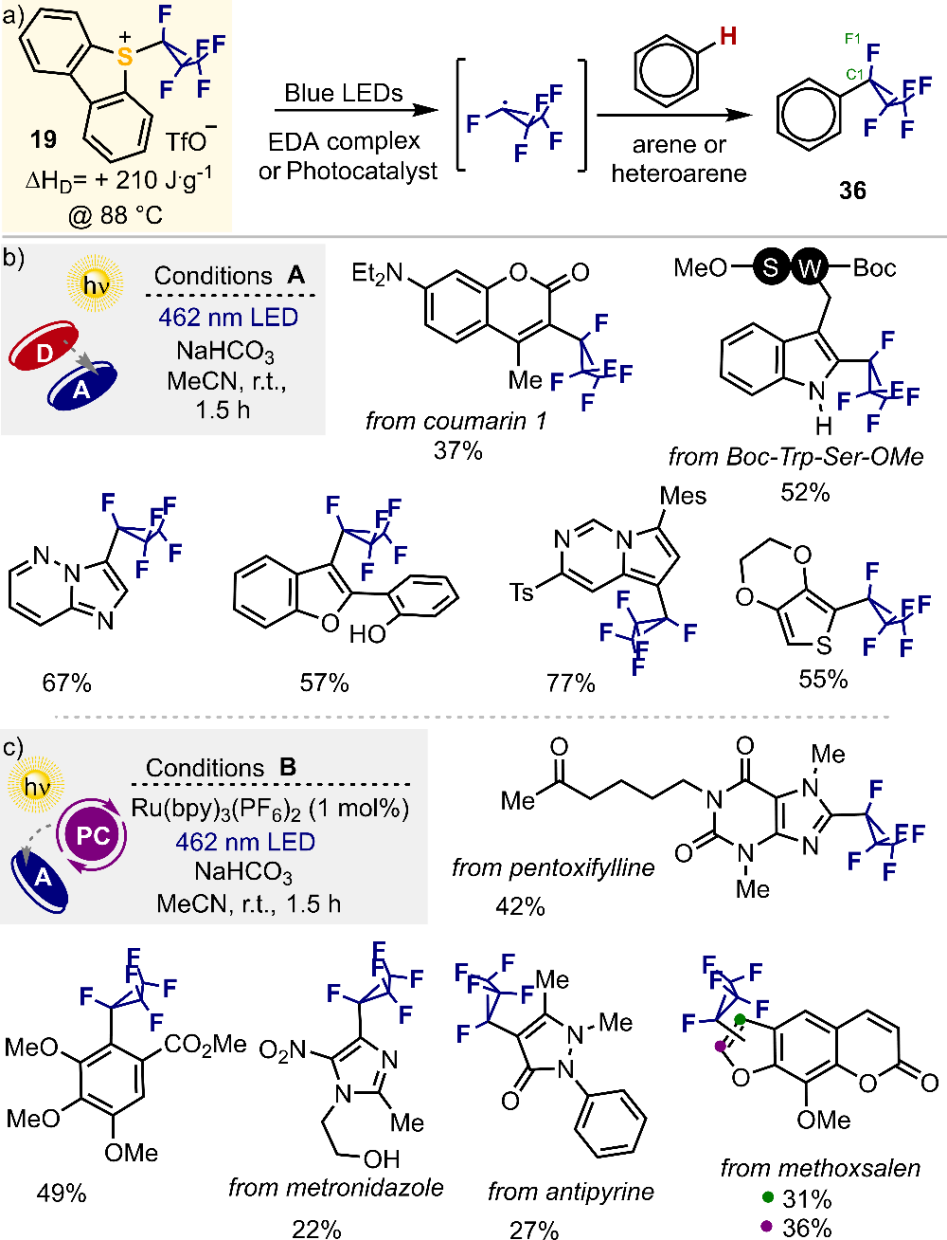
Radical Pentafluorocyclopropanations

Once available, reagent **19** was evaluated on the photoinduced C–H pentafluorocyclopropylation of (hetero)arenes through a radical mediated mechanism. Actually, two protocols were developed, as illustrated in Scheme 7. Electron-rich substrates have been found to form an electron donor–acceptor (EDA) complex after mixing the reagent and substrates. Subsequent visible light irradiation induces a single electron transfer process within the complex, leading to the mesolytic fragmentation of the S–C_cyclop_ bond, and release of the pentafluorocyclopropyl radical, which is trapped by the (hetero)aromatic substrate (Scheme 7b).^67^ In these cases, a radical chain mechanism operates. When electron-poor substrates are used, a photocatalyst -Ru(bpy)_3_(PF_6_)_2_- is needed to efficiently generate the pentafluorocyclopropyl radical (Scheme 7c). Both protocols use mild reaction conditions, and a range of functional groups such as ethers, alcohols, esters, ketones and tertiary amines are tolerated.

Interestingly, cumulative X-ray diffraction analyses of the PFCP-substituted heteroarenes revealed the preference of the cyclopropyl group to adopt a conformation in which its C1(sp^3^)–F1 bond is nearly orthogonal to the plane defined by the π-system. This is in sharp contrast to the HFIP moieties, which tend to orientate the same C1(sp^3^)–F1 bond as close to coplanarity with the heterocycle plane as the allylic-strain allows.

## 5 Skeletal Editing

During the last few decades, transition-metal-catalyzed C–H functionalization reactions have achieved recognition as one of the most highly efficient strategies for the selective introduction of functional groups into the peripherical positions of hetero- and carbocycles.^68^ Now that such strategies are reaching maturity, an even more transformative approach, known as skeletal editing, is making itself a place in the synthetic chemistry landscape.^69^ It consists on the precise incorporation, elimination or substitution of atoms from the core structure of complex architectures, enabling their quick diversification in a way that is not achievable by applying peripheral editing strategies.^20^ Although still in its infancy, methods have emerged during the last five years for the insertion or deletion of N-atoms, the incorporation of carbyne (R-C) units, and the swapping of N- or O-atoms by carbon ones.^70^

### 5.1 Nitrogen Atom Insertion

**Scheme 8:**
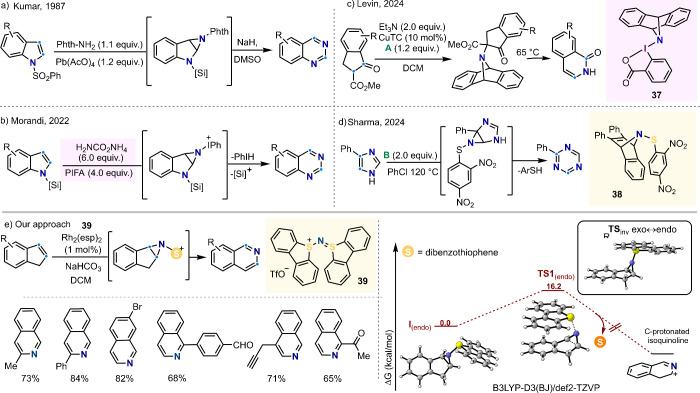
N-Atom Insertion Reactions: Available Reagents and Scope^72,74b,76^

Due to the ubiquity of nitrogen in biologically active compounds, it is not surprising that the development of protocols that allow the manipulation of atoms of this element have been the focus of intense attention.^71^ Among those, it deserves to be highlighted the work of Kumar, who already in 1987 reported the insertion of nitrogen into the pyrrolic double bond of indoles to afford the corresponding quinazoline.^72^ Subsequent studies revealed the reaction to proceed through (a) the oxidation of *N*-amino phthalimide by Pb(AcO)_4_ to deliver an acetoxyaminophthalamide, which acts as nitrene precursor;^73^ (b) the aziridination of the indole skeleton, and (c) a final ring expansion with concomitant elimination of the phthalazine unit, and delivery the final quinazoline (Scheme 8a).

The synthetic potential of this transformation was recently recognized by Morandi, who has been able to substantially expand the scope of the transformation using an *in situ* generated iodonitrene (Scheme 8b),^74^ which was obtained by oxidation of ammonium carbamate with (bis(trifluoroacetoxy)iodo)benzene (PIFA).^75^ After that seminal contribution, a series of bench stable reagents have emerged, **37**–**38**. These are able to generate nitrene equivalents under different conditions, and can be prepared in multigram scale (Scheme 8c-d).^76^

We contributed to the development of *N*-atom insertion protocols with the design and synthesis in multigram scale of the bench stable salt **39**, a reagent that is competent for the transfer of sulfonionitrene moieties to the C=C-bond of olefins under Rh-catalysis. Making use of this reactivity, we were able to gear the ring expansion of indenes into isoquinolines (Scheme 8e).^1,74a^ Mechanistically, **39** reacts with Rh_2_(esp)_2_ (esp = α,α,α′,α′-tetramethyl-1,3-benzenedipropionic acid), generating a Rh-coordinated sulfonionitrene species, which initially transfers the electrophilic nitrene moiety to olefins. After formation of the corresponding *N*-sulfonio aziridine, a ring expansion occurs via selective C–C bond cleavage and concomitant elimination of dibenzothiophene (Scheme 8e). The optimized procedure is operationally simple, tolerates a range of functional groups, including oxidation-sensitive alcohols and aldehydes, and enables the convenient preparation of valuable ^15^N-labeled products. The potential of **39** to provide alternative pathways for the selective transfer of *N*-atoms into organic scaffolds is still under evaluation in our laboratory.

### 5.2 Carbon Atom Insertion

**Scheme 9:**
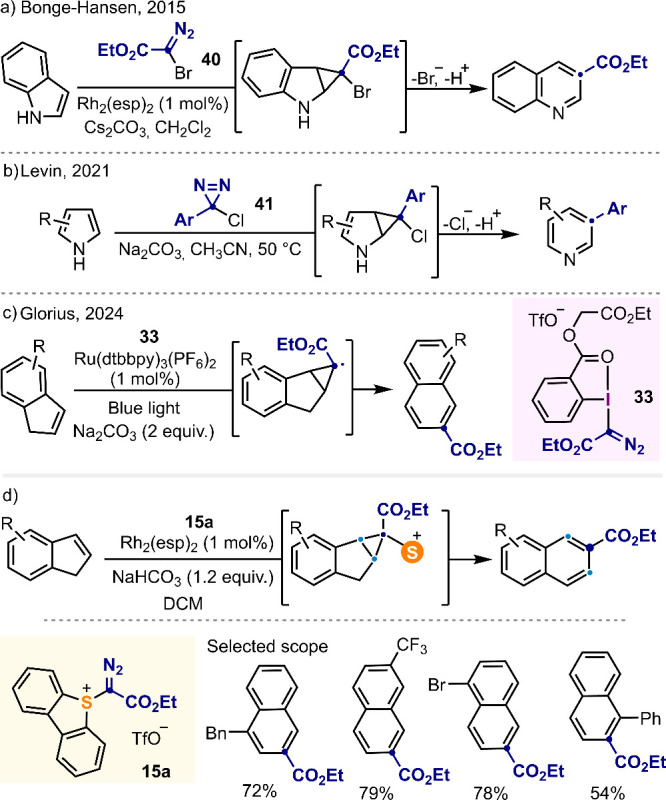
C-Atom Insertion Reactions^2,35,77−79^

The emergence of methodologies that also allow for the manipulation of carbon atoms in the core of complex structures has occurred in parallel. In his 2015 seminal paper, Bonge-Hansen reported the use of halodiazoacetates **40** as carbyne precursors under Rh-catalysis, and the use of this strategy to transform indoles into quinolines (Scheme 9a).^77^ Unfortunately, halodiazoacetates are prone to decomposition even at room-temperature, making the design of alternative equivalents of carbyne units. A rather elegant solution to this problem consists of the use of arylchlorodiazirines **41** as proposed by Levin in 2021.^78^ The increased stability of these compounds makes their handling easier than that of halodiazocompounds, but they still depict carbine reactivity. For example, indoles and pyrroles react with **41** delivering the corresponding arylpyridines and arylquinolines, respectively (Scheme 9b). Glorius has also contributed to the area with the development of a photocatalytic protocol that transforms indenes into naphthalenes employing salt **33** (Scheme 9c),^79^ which was initially described by Suero (Scheme 3c).^62^

We have contributed to the topic with the development of a Rh-catalyzed ring expansion that en-ables the transformation of indenes into naphthalenes using α-diazo sulfonium salts **15a**–**d** (Scheme 9d).^2,35^ Mechanistically, the reaction proceeds *via* initial Rh-catalyzed transfer of a sulfonio-carbene unit to olefins, delivering the corresponding cyclopropanes as mixtures of *endo*- and *exo*-diastereomers. Subsequent electrocyclic opening of the three-membered ring with concomitant elimination of dibenzothiophene delivered the final naphthalene products. Interestingly, the *endo*- and *exo*-diastereomes do not open at the same speed, making possible the isolation of the *exo*- isomers, which were characterized by X-ray crystallography.

## 6 Outlook

Our research during the past few years has demonstrated that the use of dibenzothiophenium salts provides a highly appealing alternative to I(III)-based electrophilic reagents in a series of mechanistically differentiated transformations, ranging from simple umpolung of typical nucleophiles to radical generation or atom insertion reactions. In addition, most dibenzothiophenium salts are prepared in one pot from cheap and commercially available materials, are easy to purify using traditional chromatography or crystallization methods, can be stored in multigram amounts without any special caution, and feature low oxidant character that often translates into broader functional group tolerance than the corresponding I(III)-species.

Another relevant characteristic of dibenzothiophenium salts is their enhanced thermal stability compared with their I(III) counterparts. This results in an improved safety profile, which makes the use of dibenzothiophenium salts specially recommended for scaling up processes, and offers the possibility to prepare reagents such as **39**, which has no counterpart in the I(III)-realm. I expect future research in the area to intensively exploit this fact; specifically, in combination with (already established) late stage C–H functionalization or skeletal editing techniques. As a result, new structurally well-defined reagents competent for transferring sophisticated (or seemly simple) moieties to organic substrates will be developed, expediting retrosynthetic disconnections that to date are impossible.
